# Differential microRNA expression profiling in primary tumors and matched liver metastasis of patients with colorectal cancer

**DOI:** 10.18632/oncotarget.16206

**Published:** 2017-03-15

**Authors:** Wenhua Li, Jinjia Chang, Duo Tong, Junjie Peng, Dan Huang, Weijian Guo, Wen Zhang, Jin Li

**Affiliations:** ^1^ Department of Medical Oncology, Fudan University Shanghai Cancer Center, Shanghai, China; ^2^ Department of Oncology, Shanghai Medical College, Fudan University, Shanghai, China; ^3^ Department of Colorectal Surgery, Fudan University Shanghai Cancer Center, Shanghai, China; ^4^ Department of Pathology, Fudan University Shanghai Cancer Center, Shanghai, China; ^5^ East Hospital, Tongji University School of Medicine, Shanghai, China

**Keywords:** colorectal carcinoma, microRNA, liver metastasis

## Abstract

**Background:**

Liver metastasis is common in patients with colorectal cancer (CRC), and is correlated with poor outcome. MicroRNAs (miRNAs) are small non-coding RNAs involved in cancer development and progression, but their role in CRC liver metastasis has not been extensively investigated.

**Results:**

Thirteen miRNAs were deregulated in pCRCs compared to their matched liver metastases. Seventeen miRNAs were chosen for validation, which confirmed significantly reduced expression of miR-99b-5p, miR-377 and miR-200c and increased expression of miR-196b-5p in the tissue of liver metastasis. Furthermore, miR-200c and miR-196b-5p were positively correlated with shorter overall survival in pCRC patients with liver metastasis.

**Materials and Methods:**

Firstly, affymetrix microarrays involving 1036 miRNAs were performed in two pairs of primary CRCs (pCRCs) and their matched liver metastases. Secondly, validation of the results was carried out on an independent cohort of 48 pairs of pCRCs and matched liver metastases using quantitative real-time polymerase chain reaction assay.

**Conclusions:**

We discovered a pCRC liver metastasis-specific miRNA panel including miR-377, miR-99b-5p, miR-200c and miR-196b-5p through intensive validation. These miRNAs may function as prognostic factors in patients with metastatic CRC.

## INTRODUCTION

Colorectal cancer (CRC) ranks as the third most prevalent cancer and the third leading cause of cancer mortality worldwide [1]. Liver is the most common site of metastasis. Even after resection of liver metastases, more than half the patients will not be cured due to relapse of liver metastases and emergence of other distant metastases [2, 3]. Therefore, it is critical to develop metastasis-specific molecular biomarkers for patients’ outcome prediction and treatment options.

MicroRNAs (miRNAs) are a class of endogenous, small (17–25 nucleotides), noncoding, single-stranded, evolutionarily conserved RNA molecules that regulate target gene expression by interfering with their transcription or by inhibiting translation. MiRNAs are known to be involved in the process of tumor cell growth, invasion and metastasis of CRC by regulating the expression of down-stream target genes [4–7]. Several studies reported that certain miRNAs were associated with CRC liver metastasis [4, 8–10]. For example, miR-181a and miR-200c promote liver metastases by inducing epithelial-mesenchymal transition in CRC [9, 10]. MiR-192 inhibits liver metastases by down-regulating the expression B-cell lymphoma 2, vascular endothelial growth factor A, and zinc finger E-box-binding homeobox 2 [4].

In our previous study, we found that miR-99b-5p was significantly more expressed in primary tumor than in matched liver metastasis. *In vitro*, miR-99b-5p was demonstrated to inhibit expression of mechanistic target of rapamycin (mTOR) by directly targeting its 3′ UTR. Moreover, miR-99b-5p was associated with a survival benefit in CRC patients with liver metastasis, suggesting that miR-99b-5p may serve as a predictive factor for metastasis and prognosis of CRC patients [8].

Herein, we report our metastasis-specific miRNA biomarker discovery approach which outlines the extensive miRNA difference between the primary tumor and paired liver metastasis specimens and validates newfound miRNA biomarkers in other independent cohorts of specimens. We also evaluated the correlation of the differential miRNAs expression profile with clinicopathological features and patient prognosis.

## RESULTS

### Differential miRNAs expression profile in primary tumros and matched liver metastasis tissues

We firstly performed miRNA microarray analysis in two pairs of matched pCRC and liver metastasis frozen specimens. The basic clinicopathological characteristics of these two patients were well matched. We identified 13 microRNAs whose expression levels differed between pCRC and liver metastasis tissues with a fold-change of > 1.5. Among these miRNAs, seven (miR-513a-5p, miR-181a-5p, miR-182-5p, miR-613, miR-152, miR-644a and miR-550a-5p) were up-regulated and six (miR-192-5p, miR-506-3p, miR-99b-5p, miR-29a-3p, miR-27b-3p and miR-934) were down-regulated in liver metastasis tissues compared with pCRC tissues.

**Figure 1:**
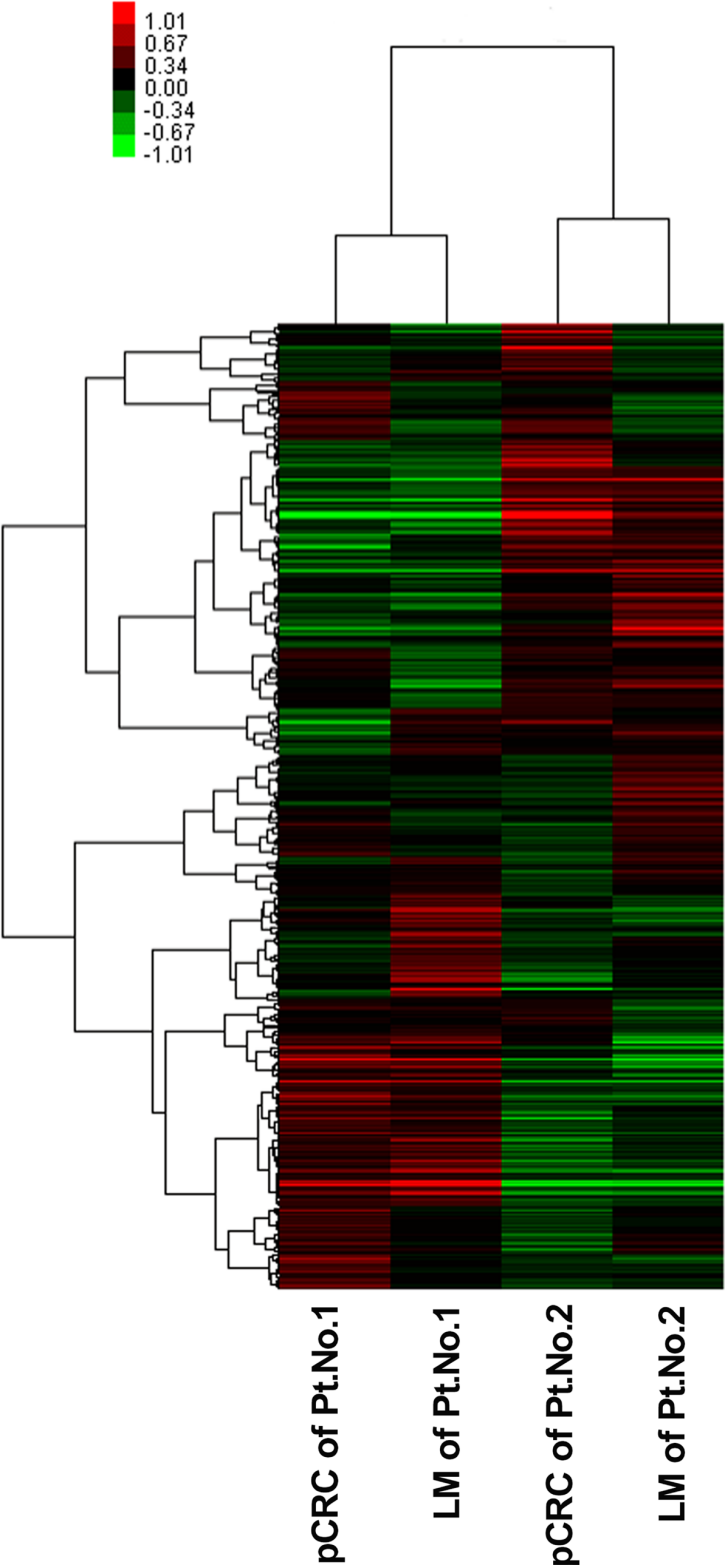
Unsupervised hierarchical cluster analysis of the 528 differentially expressed miRNAs Glue Grant Human Transcriptome Array (Affymetrix, Santa Clara, CA, USA) screened the differential expression of miRNA between primary tumor and liver metastases in two pairs of fresh tissue. Green and red represent the down-regulated and up-regulated miRNAs, respectively. LM, liver metastasis; miRNA, microRNA; pCRC, primary CRC; Pt, patient.

For the validation step, qRT-PCR assay was performed in 48 isolated pairs of pCRC and liver metastasis tissues; the clinicopathological characteristics of these 48 patients are shown in Table 1. To extend the study, we added another four microRNAs (miR-196b-5p, miR-377, miR-200c and miR-10a-5p) with fold change between 1.3 and 1.5. As a result, four miRNAs (miR-99b-5p, miR-377, miR-200c and miR-196b-5p) were found to have significant differential expression between pCRC and liver metastasis tissues. MiR-99b-5p, miR-377 and miR-200c were down-regulated, while miR-196b-5p was up-regulated in liver metastasis tissues compared to pCRC (see Table 2, Figure 1 Supplementary Figure 1).

**Table 1:** The clinicopathological characteristics of 48 colorectal cancer patients with liver metastases

Characteristics	No.	Percent
Gender		
Male	29	60.4
Female	19	39.6
Age, years		
Median (SD)	52 (28–74)	
< 60	34	70.8
≥ 60	14	29.2
Primary tumor site		
Rectum	17	35.4
Colon	31	64.6
pT		
2+3	9	18.7
4	39	81.3
pN		
0	12	25.0
1	21	43.8
2	15	31.2
Vascular invasion		
Negative	27	62.8
Positive	16	37.2
NK	5	
Perineural invasion		
Negative	27	62.8
Positive	16	37.2
NK	5	
Differentiation		
Poor/poor-moderate	12	25.0
moderate/moderate-high	36	75.0
Time of occurrence of liver metastasis		
Synchronous	43	89.6
Metachronous	5	10.4
Combined with other metastases		
No	41	85.4
Yes	7	14.6

Abbreviations: NK, not known; NR, not reached; SD, standard deviation.

**Table 2:** Expression level of miRNAs in the primary tumor and matched liver metastases of 48 colorectal cancer patients with liver metastases

miRNA	Tumor status	Median	SEM	Foldchange (primary/ metastasis)	*p* value
miR-513a-5p	Primary	28.9670	7.87524	0.54	0.113
	Metastasis	53.6298	15.04555		
miR-181a-5p	Primary	2.2210	1.11954	0.51	0.188
	Metastasis	4.3494	0.99962		
miR-182-5p	Primary	19.8616	4.40714	1.48	0.253
	Metastasis	13.3865	4.96276		
miR-613	Primary	64.2181	41.80958	1.20	0.877
	Metastasis	53.7233	52.13662		
miR-152	Primary	31.6605	18.05150	9.32	0.114
	Metastasis	3.3972	0.93292		
miR-644a	Primary	18.0385	9.14903	0.26	0.313
	Metastasis	69.2121	54.83062		
miR--550a-5p	Primary	179.4224	87.74360	0.51	0.291
	Metastasis	354.2439	219.88422		
miR-196b-5p	Primary	8.8372	4.05013	0.18	0.046*
	Metastasis	49.5967	20.74880		
miR-10a-5p	Primary	5.4569	2.19123	0.16	0.062
	Metastasis	33.9821	16.66163		
miR-192-5p	Primary	4304.9628	1690.66343	4.77	0.051
	Metastasis	903.1332	275.60286		
miR-506-3p	Primary	11.2938	5.02323	1.07	0.908
	Metastasis	10.5453	6.00231		
miR-99b-5p	Primary	8.8244	2.75573	6.77	0.007*
	Metastasis	1.3043	0.29901		
miR-29a-3p-	Primary	1.3543	0.90819	4.44	0.169
	Metastasis	0.3049	0.18774		
miR-27b-3p	Primary	2.6432	0.61204	0.07	0.170
	Metastasis	39.7780	26.64356		
miR-377	Primary	92.8067	40.17213	433.47	0.026*
	Metastasis	0.2141	0.13345		
miR-200c	Primary	13.4106	3.51479	3.77	0.009*
	Metastasis	3.5561	0.95874		
miR-934	Primary	15.6664	11.61170	1.78	0.515
	Metastasis	8.8113	2.47346		

Abbreviations: miRNA, microRNA; SEM, standard error of the mean. *p ≤ 0.05.

### MiR-377 and miR-99b-5p expression levels serve as invasive biomarkers in pCRC patients with liver metastasis

To explore the clinical significance of these liver metastasis-specific miRNAs, we further validated the correlation between microRNAs and clinicopathological characteristics. Three up-regulated miRNAs (miR-99b-5p, miR-377 and miR-200c) and one down-regulated miRNA (miR-196b-5p) in pCRC compared to liver metastasis tissues were analyzed. As shown in Table 3, increased miR-377 expression level was associated with advanced lymph node stage (*p* = 0.043). Likewise, decreased miR-99b-5p expression was positively correlated with perineural invasion in pCRC (*p* = 0.043).

**Table 3:** Relationship between the expression level of miRNAs in the primary tumor and clinicopathologic parameters in 48 colorectal cancer patients with liver metastases

	No.	Percent	miR-377			miR-200c			miR-99b-5p			miR-196b-5p		
Characteristics			Mean	SEM	*p* value	Mean	SEM	*p* value	Mean	SEM	*p* value	Mean	SEM	*p* value
**Gender**														
Male	29	60.4	133.69	64.193	0.138	9.99	2.805	0.233	9.64	3.998	0.719	12.22	6.537	0.307
Female	19	39.6	30.41	22.445		18.64	7.765		7.58	3.466		3.68	2.070	
**Age, years**														
Median (SD)	52 (28–74)													
< 60	34	70.8	41.79	19.491	0.194	13.36	4.735	0.983	8.28	3.613	0.762	3.07	1.098	0.16
≥ 60	14	29.2	216.69	126.472		13.53	3.864		10.15	3.668		22.83	13.215	
**Primary tumor site**														
Rectum	17	35.4	41.92	25.288	0.354	19.59	9.081	0.319	15.34	6.992	0.178	4.85	2.225	0.472
Colon	31	64.6	120.71	60.452		10.02	2.183		5.25	1.709		11.02	6.156	
**pT**														
2+3	9	18.7	64.61	32.559	0.74	9.70	2.394	0.617	3.86	1.710	0.392	19.13	15.913	0.457
4	39	81.3	99.31	48.982		14.27	4.292		9.97	3.352		6.46	3.446	
**pN**														
Negative	12	25.0	9.23	3.215	0.043*	15.88	5.763	0.69	12.06	6.051	0.503	13.89	12.014	0.477
Positive	36	75.0	120.67	52.912		12.59	4.308		7.74	3.103		7.15	3.732	
**Vascular invasion**														
Negative	27	62.8	85.06	62.303	0.673	12.37	3.394	0.525	12.91	4.688	0.088	4.17	1.549	0.216
Positive	16	37.2	124.78	59.972		17.56	8.888		4.17	1.638		19.34	11.657	
NK	5													
**Perineural invasion**														
Negative	27	62.8	35.52	18.413	0.151	13.30	3.371	0.742	13.45	4.710	0.043*	9.61	5.442	0.953
Positive	16	37.2	208.38	113.022		15.99	8.959		3.25	0.961		10.17	8.117	
NK	5													
**Differentiation**														
Poor/poor-moderate	12	25.0	124.21	79.322	0.657	7.67	2.146	0.352	3.61	1.083	0.074	12.81	10.703	0.577
Moderate/moderate-high	36	75.0	82.34	47.093		15.32	4.606		10.56	3.623		7.51	4.143	
**Time of occurrence of liver metastasis**														
Synchronous	43	89.6	99.78	44.722	0.616	10.84	2.265	0.432	9.08	3.048	0.786	9.77	4.505	0.507
Metachronous	5	10.4	32.86	20.699		35.48	28.185		6.60	4.063		0.85	0.300	
**Combined with other metastases**														
No	41	85.4	88.66	43.422	0.806	11.12	2.401	0.466	9.65	3.205	0.475	7.08	3.650	0.543
Yes	7	14.6	117.11	113.770		26.84	20.083		4.00	1.473		19.15	18.443	

Abbreviations: NK, not known; SD, standard deviation; SEM, standard error of the mean.

### MiR-200c and miR-196-5p expression levels serve as prognostic biomarkers in pCRC patients with liver metastasis

**Figure 2:**
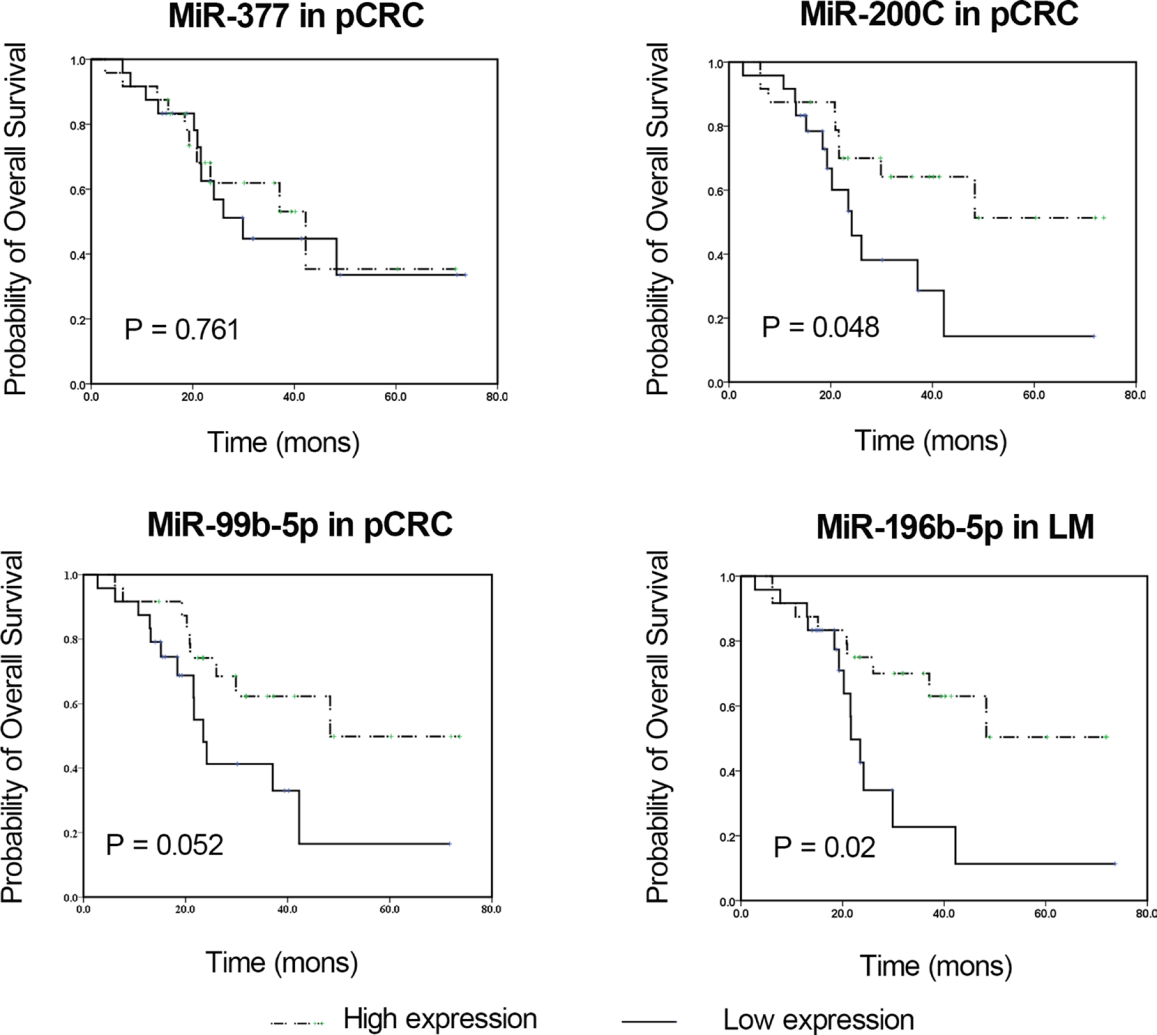
Kaplan-Meier survival curves of CRC patients subdivided by miRNAs levels in pCRC and LM tissues from CRC patients Overall survival analysis based on miR-377, miR-200c, miR-99b-5p and miR-196b-5p in pCRC or LM tissues are shown. The *p* value was determined by log-rank test. LM, liver metastasis; miRNA, microRNA; pCRC, primary CRC.

We tested the association between miRNA expression and overall survival by performing survival analysis. As shown in Figure 2 and Table 4, patients with elevated expression levels of miR-200c in pCRC and miR-196b-5p in liver metastases had significantly longer survival by Kaplan-Meier analysis (*p* = 0.048 and *p* = 0.02, respectively). Notably, higher expression of miR-99b-5p in pCRC also showed a better trend of survival, although statistical significance was not reached (*p* = 0.052).

**Table 4:** Relationship between the expression level of miRNAs and overall survival in 48 colorectal cancer patients with liver metastases

				95% CI						95% CI	
Primary tumor		Median OS	SEM	Lower bound	Upper bound	Liver metastases		Median OS	SEM	Lower bound	Upper bound
**miR-377**	Low	29.867	5.509	19.069	40.664	**miR-377**	Low	24.133	13.966	0	51.506
	High	42.233	11.776	19.152	65.314		High	37.067	NR	NR	NR
	*p* value	0.761					p value	0.417			
**miR-200c**	Low	24.133	3.290	17.684	30.582	**miR-200c**	Low	26.033	5.702	14.858	37.209
	High	NR	NR	NR	NR		High	48.3	12.092	24.599	72.001
	*p* value	0.048*					p value	0.266			
**miR-99b-5p**	Low	23.467	2.212	19.131	27.802	**miR-99b-5p**	Low	23.467	1.594	20.343	26.59
	High	48.300	NR	NR	NR		High	48.3	12.096	24.592	72.008
	*p* value	0.052					p value	0.098			
**miR-196b-5p**	Low	23.467	1.71	20.115	26.819	**miR-196b-5p**	Low	21.633	1.686	18.329	24.937
	High	42.233	NR	NR	NR		High	NR	NR	NR	NR
	*p* value	0.116					p value	0.02*			

Abbreviations: CI: confidence interval; miRNA, microRNA; NR: not reached; OS, overall survival; SEM, standard error of the mean. *p ≤ 0.05.

## DISCUSSION

Patients with liver metastasis in CRC were with a poor prognosis and early recurrence. Previous researches in the fields of molecular and genetic studies have contributed to our understanding of CRC liver metastasis in the cellular level [11, 12]. However, heterogeneity of research methods as well as tumor tissue acquisition limited the diagnostic and prognostic role of specific genes in metastases.

Recent findings have highlighted the role of miRNAs in the tumorigenesis of CRC cells acting as oncogenes or tumor suppressor genes [13, 14]. Similar studies have been performed to gain better insight into genomic changes involved in pCRC initiation and progression [15]. However, there are few studies of miRNA expression accounting for distant metastasis of pCRC. In this study, we aim to identify a differential microRNA expression profile in pCRC and their matched liver metastasis.

In this study, expression profiling of 1036 miRNAs based on an RNA array platform in clinical samples of pCRC tissues with matched liver metastasis tissues was carried out to identify advanced CRC signatures and to use miRNA expression profiles to classifying CRC with liver metastasis. The confirmation of the array data was done by using qRT-PCR. The results, by microarray analysis in two paired colorectal cancers, showed 17 miRNAs were dysregulated in liver metastasis tissues compared with pCRC tissues. These differentially expressed miRNAs were further validated by qRT-PCR in 48 isolated pairs of pCRC and liver metastasis tissues, and four miRNAs (miR-99b-5p, miR-377, miR-200c and miR-196b-5p) were found to have significant differential expression between pCRC tissues and liver metastasis tissues. While examining the potential clinical impact of these four liver metastasis-specific miRNAs, we noticed that up-regulation of miR-200c in pCRC tissues and miR-196b-5p in liver metastasis tissues were associated with significantly longer survival by Kaplan-Meier analysis (*p* = 0.048 and *p* = 0.02, respectively).

We previously demonstrated that miR-99b-5p is a tumor suppressing miRNA in metastatic CRC, and that its suppressive effects are mediated chiefly by suppressing mTOR expression [8]. MiR-196b was reported to be highly expressed in CRC tissues and was correlated with rapid progression and poor prognosis [16, 17]. MiR-196b was also a predictive factor in palliative chemotherapy and neoadjuvant radiochemotherapy [18, 19]. Similar to our findings, high expression of miR-200c was observed in liver metastasis [10] and its expression in serum was reported to be a prognostic factor in patients with CRC [20]. It has been found that miR-200c could mediate epithelial-mesenchymal transition in liver metastases by regulating the TGF-TGFegu signal pathway [21, 22]. MiR-377 was a novel biomarker for poor prognosis in gastrointestinal cancer, which induced tumorigenesis by targeting multiple tumor-suppressor genes [23].

Our study is distinct from previous studies in several aspects. First, we directly compared pCRC and matching liver metastasis tissues from the same patients, which avoid heterogeneity due to distinct miRNA from different patients. Second, to improve the predictive value of the miRNA markers, we developed a miRNA-based classifier panel rather than just suggesting each miRNA as a predictor.

In conclusion, four miRNAs (miR-99b-5p, miR-377, miR-200c and miR-196b-5p) were found to be differently expressed between primary tumors of patients with CRC and their matched liver metastases. Moreover, we nominate the candidate microRNAs, miR-200 and miR-196b-5p as potential prognostic markers. Our study suggested that these miRNAs could hopefully serve as metastasis-specific miRNA biomarkers for the management of patients with CRC.

## MATERIALS AND METHODS

### Patient samples

The fresh-frozen/formalin-fixed, paraffin-embedded primary CRC and matched corresponding liver metastatic tissues were collected from 2004 to 2012 in the Department of Colorectal Cancer, Fudan University Shanghai Cancer Center. The tissue bank of the hospital stored the specimens. The study was approved by the Ethics Committee of Fudan University Shanghai Cancer Center and written informed consent was obtained from all patients.

### RNA extraction and miRNA array hybridization

Total RNAs from two CRC patients with liver metastasis were analyzed by microarray. Total RNA was isolated using Trizol (Invitrogen, Carlsbad, CA, USA). RNA was labeled and hybridized to Glue Grant Human Transcriptome Array (Affymetrix, Santa Clara, CA, USA) [24–26] according to manufacturer's instructions. Command Console^®^ Software version 3.1 was used for microarray analysis. A total of 1036 miRNAs were covered using miRBase version 18.0.

### Quantitative real-time polymerase chain reaction

miR-513a-5p, miR-181a-5p, miR-182-5p, miR-613, miR-152, miR-644a, miR-550a-5p, miR-192-5p, miR-506-3p, miR-99b-5p, miR-29a-3p, miR-27b-3p, miR-934, miR-196b-5p, miR-200c, miR-377, miR-10a-5p and RNU6B (internal control) specific cDNA syntheses were carried out using the miScript Reverse Transcription Kit (Qiagen, Hilden, Germany) according to the manufacturer's protocols. The expression level of miRNAs above was measured using TaqMan miRNA assays (Applied Biosystems, Carlsbad, CA, USA). U6 was used as an endogenous control and the fold change was calculated using the 2^−ΔΔCt^ method.

### Statistical analysis

The data were analyzed with SPSS version 18.0. The student *t*-test and one factor analysis of variance were used for analysis as appropriate. Kaplan-Meier method and log-rank test were used to estimate the survival. *P* < 0.05 was considered statistically significant.

## SUPPLEMENTARY MATERIALS FIGURES



## Abbreviations

CRC: colorectal cancer
miRNA: microRNA
mTOR: mechanistic target of rapamycin
pCRC: primary colorectal cancer
qRT-PCR: quantitative real-time polymerase chain reaction
