# Supplementary material for: Differential microRNA expression profiling in primary tumors and matched liver metastasis of patients with colorectal cancer
Source: Oncotarget. 2017 Mar 15;8(22):35783–91. doi: 10.18632/oncotarget.16206 (PMC5482617; doi:10.18632/oncotarget.16206)
Supplement: Supplementary file 1 [file oncotarget-08-35783-s001.pdf]

Differential microRNA expression profiling in primary tumors and matched liver metastasis of patients with colorectal cancer

Supplementary Materials

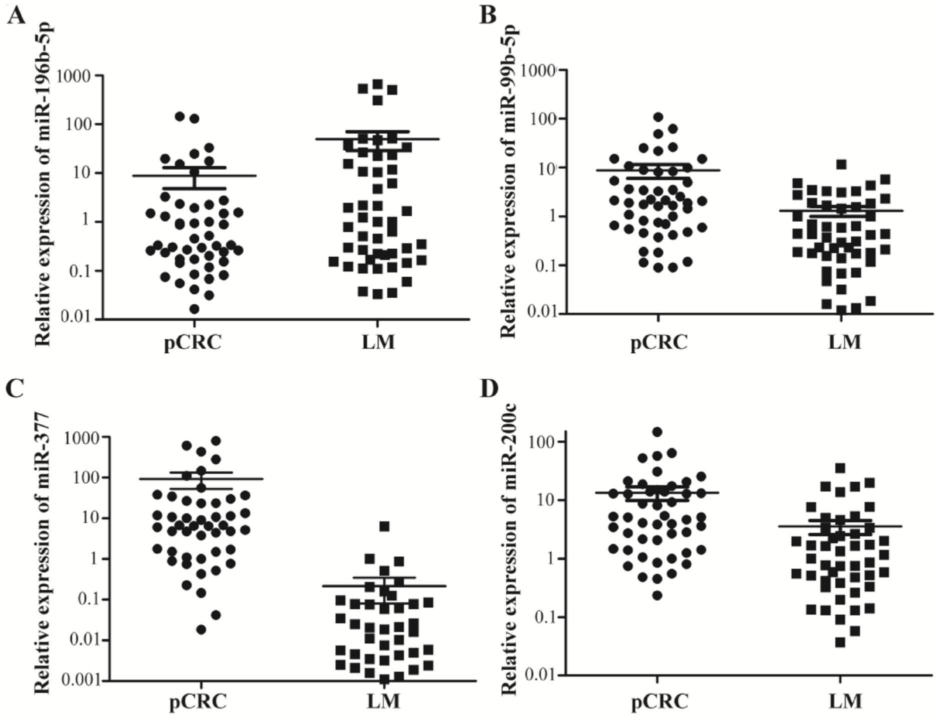

Supplementary Figure 1: Differential expression of miRNAs between primary tumor and liver metastasis tissues in 48 colorectal cancer patients. miRNA-196b-5p (**A**, $P = 0.046$) was up-regulated in liver metastasis tissues compared to primary colorectal cancer, while miR-99b-5p (**B**, $P = 0.007$), miR-377 (**C**, $P = 0.026$) and miR-200c (**D**, $P = 0.009$) were down-regulated. LM, liver metastasis; miRNA, microRNA; pCRC, primary CRC.
